# The contribution of marine aggregate‐associated bacteria to the accumulation of pathogenic bacteria in oysters: an agent‐based model

**DOI:** 10.1002/ece3.2467

**Published:** 2016-09-24

**Authors:** Andrew M. Kramer, J. Evan Ward, Fred C. Dobbs, Melissa L. Pierce, John M. Drake

**Affiliations:** ^1^ Odum School of Ecology University of Georgia Athens GA USA; ^2^ Center for Ecology of Infectious Diseases University of Georgia Athens GA USA; ^3^ Department of Marine Sciences University of Connecticut Groton CT USA; ^4^ Department of Ocean, Earth and Atmospheric Sciences Old Dominion University Norfolk VA USA

**Keywords:** aquatic pathogens, attached bacteria, marine snow, organic aggregates, *Vibrio* species, waterborne disease

## Abstract

Bivalves process large volumes of water, leading to their accumulation of bacteria, including potential human pathogens (e.g., vibrios). These bacteria are captured at low efficiencies when freely suspended in the water column, but they also attach to marine aggregates, which are captured with near 100% efficiency. For this reason, and because they are often enriched with heterotrophic bacteria, marine aggregates have been hypothesized to function as important transporters of bacteria into bivalves. The relative contribution of aggregates and unattached bacteria to the accumulation of these cells, however, is unknown. We developed an agent‐based model to simulate accumulation of vibrio‐type bacteria in oysters. Simulations were conducted over a realistic range of concentrations of bacteria and aggregates and incorporated the dependence of pseudofeces production on particulate matter. The model shows that the contribution of aggregate‐attached bacteria depends strongly on the unattached bacteria, which form the colonization pool for aggregates and are directly captured by the simulated oysters. The concentration of aggregates is also important, but its effect depends on the concentration of unattached bacteria. At high bacterial concentrations, aggregates contribute the majority of bacteria in the oysters. At low concentrations of unattached bacteria, aggregates have a neutral or even a slightly negative effect on bacterial accumulation. These results provide the first evidence suggesting that the concentration of aggregates could influence uptake of pathogenic bacteria in bivalves and show that the tendency of a bacterial species to remain attached to aggregates is a key factor for understanding species‐specific accumulation.

## 1 Introduction

Bivalve mollusks are important residents of many nearshore environments. They often dominate bottom communities, forming large clustered populations that are both ecologically and commercially important (Dame, 1993; FAO 2014). Suspension‐feeding bivalves in general, and oysters in particular, have been studied extensively, and their ability to pump and process large volumes of water per unit time is well documented (e.g., 3–8 L water hr^−1^ g‐dry weight^−1^; Newell, 1988; Riisgård, 1988; Newell, Pilskaln, Robinson, & MacDonald, 2005; Cranford, Ward, & Shumway, 2011). As a result of their feeding activities, dense populations of bivalves significantly impact the chemical and biological processes of both the benthic and pelagic realms (Dame, 1993; Heinonen, Ward, & Holohan, 2007; Newell, 2004; Prins, Smaal, & Dame, 1997).

The processing of large volumes of water by bivalves leads to the intake and concentration of substantial numbers of microorganisms. These microorganisms, however, are captured with varying efficiency. Particles greater than ca. 5 μm are captured with about 100% efficiency, whereas smaller ones are captured with an efficiency that decreases asymptotically with decreasing particle size (Riisgård, 1988). Although a few bivalve species (e.g., ribbed mussel, *Geukensia demissa*) are able to capture 1‐μm particles with approximately 40% to 50% efficiency (Kreeger & Newell, 1996; Langdon & Newell, 1989), most species capture particles smaller than 1 μm at an efficiency <20% (Kach & Ward, 2008; Møhlenberg & Riisgård, 1978; Palmer & Williams, 1980; Riisgård, 1988; Ward & Shumway, 2004).

The exception to this rule occurs when particles and cells <5 μm are incorporated into the matrix of marine aggregates. Aggregates (aka, flocs, organic detritus) are agglomerations of suspended particulate matter formed by physical, chemical, and biological processes (Alldredge & Silver, 1988; Azam & Malfatti, 2007; Jackson, 1990). Aggregates range in size from 10s to 1,000s of μm (0.01–10 mm), with those larger than 500 μm (0.5 mm) being classified as marine snow (Alldredge & Silver, 1988). Therefore, they and their constituent particles can be captured with an efficiency of about 100% (Ward & Shumway, 2004), much higher than that of individual particles measuring <5 μm. For example, the ingestion rate of suspended polystyrene beads and bacteria (0.1–1.0 μm) by several species of bivalves is low, but increases significantly when the same particles are incorporated into aggregates (Kach & Ward, 2008; Ward & Kach, 2009).

The concentration of aggregates in the marine environment varies over multiple temporal (tidal, daily, seasonal) and spatial (estuarine, coastal, deep sea) scales (Newell et al., 2005; Shanks, 2002; Simon, Grossart, Schweitzer, & Ploug, 2002). Typically, macroaggregate concentrations in coastal waters range from 1 to 1,000/L (Simon et al., 2002). During certain times of the year, a large proportion (>70%) of natural particulates of varying size and quality form aggregates mixed with high molecular weight substances (Alldredge, Passow, & Logan, 1993; Passow, Alldredge, & Logan, 1994; Stolzenbach, 1993; Syvitski, Asprey, & Leblanc, 1995; Verney, Lafite, & Brun‐Cottan, 2009). The aggregation of suspended particles is an important process that increases the downward vertical transport of material to the benthos (Crocker & Passow, 1995; Kiørboe, Andersen, & Dam, 1990; Passow & Wassmann, 1994).

Aggregates have a complex three‐dimensional structure that is physically, chemically, and biologically distinct from the surrounding water (Alldredge, 2000; Ploug, 2001; Silver, Shanks, & Trent, 1978). In particular, bacterial community structure and diversity often differ significantly from that of the water (Azam & Long, 2001; Crump, Armbrust, & Baross, 1999; Kiørboe, Tang, Grossart, & Ploug, 2003; Kramer, Lyons, Dobbs, & Drake, 2013), with enzyme activities and metabolism significantly higher than for free‐living (unattached) communities (Azam & Malfatti, 2007; Grossart, Tang, Kiørboe, & Ploug, 2007; Lyons & Dobbs, 2012). In addition to bacteria, marine aggregates may be colonized by protozoans, phytoplankton, and metazoans in concentrations that are 100–10,000 times greater than in the surrounding water (Lampitt, Wishner, Turley, & Angel, 1993; Lyons et al., 2007; Shapiro et al., 2013; Simon et al., 2002). Thus, aggregates are not only *concentrators* of microscopic organisms, but also *transporters* that move biological species throughout the water column.

The factors that concentrate and transport naturally occurring bacteria on aggregates may also facilitate the movement of disease‐causing agents, including pathogens that infect bivalves and other animals (Lyons et al., 2007; Shapiro et al., 2013). Bacterial pathogens found within marine aggregates include the following: *Vibrio cholerae, V. parahaemolyticus, V. vulnificus, V. alginolyticus, Aeromonas hydrophila, Pseudomonas aeruginosa, Escherichia coli,* and *Mycobacteria* sp. (Lyons et al., 2007; Venkateswaran et al., 1990). Some of these bacteria, when accumulated in high concentrations by commercially important bivalves (e.g., oysters) and subsequently ingested, may cause illness and death in humans (Morris & Acheson, 2003).

The association between vibrios and aggregates can be partially attributed to the bacteria's preference for environments rich with organic matter (Tinta, Kogovšek, Malej, & Turk, 2012). In general, vibrios live an attached lifestyle, can be associated with phytoplankton and zooplankton, and are common symbionts with a number of metazoans (Thompson, Iida, & Swings, 2004 and references therein). Field collections using molecular fingerprinting have shown Vibrionales in high abundances in marine snow, and absent or in low abundances in ambient waters (Vojvoda et al., 2014). Despite the depth of knowledge concerning the ecology and lifestyle of vibrios, few investigators have quantified pathogen concentrations on aggregates collected in the field. Notably, Lyons et al. (2007) detected “mesophilic pathogenic bacteria” (predominantly culturable *Vibrio* spp.) at concentrations between 10^0^ and 10^5^ colony‐forming units (CFUs) per ml of aggregates. Their values represent samples from four habitats over the course of 8 months. The majority of data in this regard, however, are based on laboratory experiments that arguably tend to enrich microorganisms. For example, Froelich, Ayrapetyan, and Oliver (2013) reported *Vibrio vulnificus* concentrations ranging between 6 × 10^4^ and 2 × 10^5^ CFUs/ml of aggregates, while Lyons et al. (2010) found vibrios present at >10^6^ CFUs/ml and *Escherichia coli* at approximately 10^2^ CFUs/ml of aggregates.

Two physical attributes of aggregates could contribute to their role in transporting pathogen into bivalves. First, they are much larger, and therefore sink faster, than their smaller constituent particles (Kiørboe et al., 1990; Waite, Safi, Hall, & Nodder, 2000). Consequently, benthic bivalves are exposed to a supply of marine aggregates and the pathogens they contain. Second, because of their larger size, aggregates are readily captured by suspension‐feeding bivalves, whereas smaller, unattached cells are captured at a lower efficiency. These effects have been demonstrated in previous studies where the rate at which *E. coli* and *Vibrio vulnificus* are ingested by shellfish increases significantly when the bacterial cells are incorporated into aggregates (Froelich et al., 2013; Kach & Ward, 2008). Importantly, aggregates enhance the transmission of certain bivalve diseases such as dermo disease of oysters, *Perkinsus marinus* (Allam et al., 2013).

Given the ecological and human‐health connections among marine aggregates, pathogenic bacteria, and bivalves, it is important to understand how these three components interact to mediate pathogen accumulation and associated risk to humans. Whereas the studies cited above suggest that effects of aggregates on pathogen accumulation in shellfish may be substantial, none addresses how the concentration of pathogenic bacteria and the abundance of marine aggregates affect pathogen accumulation or how much individual variation is to be expected among bivalves. To address these questions, we developed a model for presence of bacteria on aggregates and the subsequent uptake by *Crassostrea* spp., accounting for these biologically important details. Our model describes qualitative differences in the relative contributions of unattached and aggregate‐associated bacteria to pathogen load and makes testable predictions. The results provide insight into the role of aggregates in bacterial uptake and highlight the key traits and processes that mediate ingestion of bacteria by oysters. When combined with additional information on these processes, this model provides a framework for predictive modeling of specific pathogens.

## 2 Methods

### 2.1 Model framework

Consumption of unattached and aggregate‐associated bacteria by oysters was simulated as an agent‐based model of a stochastic, discrete‐time process with time steps of 1 min. The model was intended to generate insight into the qualitative dynamics of focal bacteria that represent human pathogenicity, such as *Vibrio cholerae* or *V. vulnificus*. These focal bacteria are hereafter referred to as “pathogen” to distinguish them from the overall bacterial community of which they are a part (Kiørboe, 2003; Kramer et al., 2013). The model consisted of 441 oysters, each 100 mm in shell height and arrayed in a uniform grid (21 × 21 cells), providing a realistic representation of a flat oyster bed or oyster cage approximately 2 m by 2 m square. For the current application spatial location on the grid is uninformative, but this model structure allows for future extension to consider water currents, advective flow or food depletion. In each time step, oysters processed a fixed volume of water containing a random collection of particles including bacterial cells and aggregates, the size and pathogen load of which was drawn from statistical distributions. Oysters rejected a proportion of incoming pathogens as pseudofeces, with the proportion rejected varying with aggregate concentration, a proxy for total suspended particulate matter (SPM in mg/L; Newell et al., 2005). Pathogen cells associated with aggregates were assumed to be captured with 100% efficiency, while unattached pathogens were captured with much lower efficiency (as described below). Accumulated pathogens were lost from the oyster at a constant rate due to digestion and mortality. Using this approach, the average number of pathogen cells and the proportion arriving from unattached and aggregate‐associated routes were simulated for a variety of aggregate concentrations and pathogen concentrations. Parameter values are described below and summarized in Table 1.

**Table 1:** Parameter values

Definition	Units	Default value^a^
Oyster length	mm	100
Clearance rate	L/min	0.22^a^
Capture of aggregate‐associated pathogen	–	1
Capture of unattached pathogen	–	0.16^b^
Bacterial loss rate in oyster	min^−1^	0.0014^c^
Proportion of material rejected as pseudofeces	–	0.61–0.67^d^
Concentration of unattached pathogen	cells/ml	10, 100, 1,000, 10,000^e^
Macroaggregate concentrations	L^−1^	50–500
Macroaggregate radii	mm	0.1–10
Microaggregate radius	mm	0.075

a Based on oyster length (Ren et al., 2000).

b Based on single bacterial cells of vibrios (Ward & Shumway, 2004).

c Based on full clearance of gut in 12 hr.

d See Methods.

e Kramer et al. 2013.

### 2.2 Feeding parameters

Simulated oysters were assumed to clear a constant volume (*V*) of water per time step (L/min) related to their length as *V* = 0.016 × length^1.46^/60, where length is in units of millimeters (Ren, Ross, & Schiel, 2000). Because many endogenous and exogenous factors can influence oyster clearance rate (Newell & Langdon, 1996), we used a maximum rate for warm‐water months, making this assumption a scenario of maximum accumulation. The clearance rate used in the model (0.22 L/min) is consistent with that recommended in a recent review of oyster filtration rate (Ehrich & Harris, 2015) and the dry tissue mass of an oyster of 100 mm in shell height (Dame 1972). The summertime rate is most relevant for pathogens, such as vibrios, that are typically more abundant at warmer water temperatures. Including natural variation in clearance rate would reduce the total load of pathogen after a given time period, but does not affect the relative quantities of unattached and aggregate‐associated bacteria consumed. Whereas all the pathogen cells on an aggregate were assumed to be captured by model oysters, only 16% of unattached cells were captured (e.g., Ward & Shumway, 2004). The loss of consumed pathogens from the oyster was the cumulative effect of pathogen growth, digestion, and other loss processes at a rate of 0.0014 min^−1^. This estimated rate assumed constant loss leading to gut clearance in 12 hr and was independent of gut contents, and hence not a rate per bacterial cell. Reported gut retention times in bivalves vary greatly, from <2 hr to >27 hr depending on species, and the quantity and quality of material ingested (see Brillant & MacDonald, 2000 for review). For oysters, retention times are not well established, but values between 8 and 12 hr have been reported for some species (e.g., Chaparro, Soto, Thompson, & Concha, 2001). Therefore, we used 12 hr as a notional time for gut clearance of pathogens.

### 2.3 Pseudofeces production

Simulated oysters rejected a proportion of all incoming material depending on the concentration of aggregates in the water. Pseudofeces production increases with increasing SPM until a saturating seston level is reached, beyond which feeding is inhibited (Barillé, Prou, Héral, & Razet, 1997; Deslous‐Paoli et al., 1992; Hawkins et al., 1998). Here, the relationship between SPM and aggregate concentration was estimated using linear regression from data collected by Shanks (2002; reported as total suspended solids, data from this Fig. 4 with the outlier at ~8 mg/L excluded). Data from Shanks (2002) were also used for the distribution of aggregate sizes and represent high concentrations of SPM (15–20 mg/L). After estimating the SPM from the aggregate concentration, the pseudofeces production for each level of SPM was estimated using a saturating function (Fig. S1, Hawkins et al., 1998; Mafra, Bricelj, & Ward, 2009; Ward & Holohan unpublished). The high level of SPM in the empirical dataset (Shanks, 2002) linking SPM to aggregate concentration resulted in high levels of pseudofeces production (Table 1), with unattached and aggregate‐associated pathogens being rejected at equal rates. There is no direct experimental evidence tracking the propensity of unattached bacteria to be rejected with pseudofeces; however, the feeding process involves the disaggregation of material by the gills and labial palps, and mixing of material from multiple sources (e.g., different gill lamellae, different palp lamellae) before the production of pseudofeces (Ward, 1996). This mixing makes it likely that pseudofeces production affects both unattached and aggregate‐associated bacteria.

### 2.4 Aggregate size distribution

**Figure 1:**
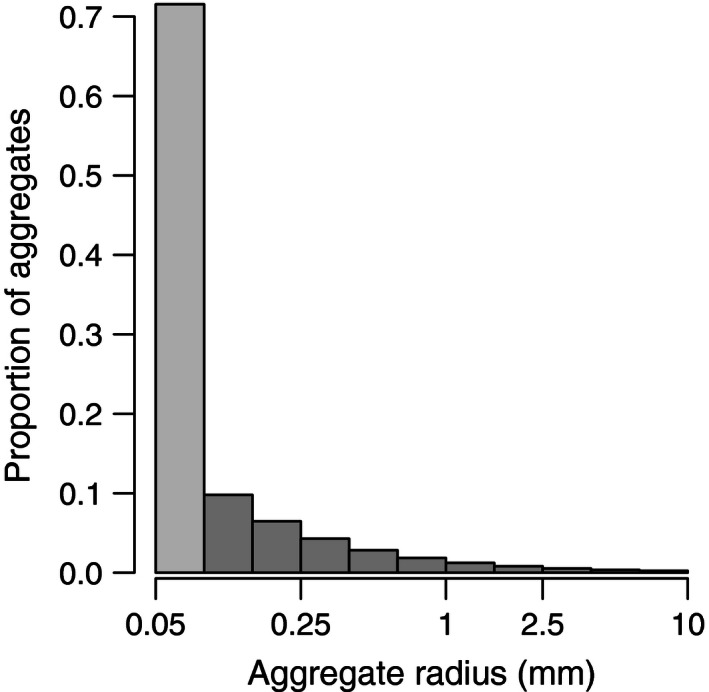
Estimated aggregate size distribution. The size distribution of aggregates was obtained from fitting an exponential model to data from Shanks (2002). Data were combined from sampling across several dates that varied in season and aggregate concentration. The midpoints of these bins were used to simulate pathogen accumulation on different‐sized aggregates. The light gray‐colored bar represents the estimated abundance of microaggregates (see text)

The relative abundance of aggregates of different sizes was estimated from empirical observations (Shanks, 2002). The data from six sampling dates (Fig. 5 in Shanks, 2002) were pooled and a single regression fit on a log–log scale (*p* = 1e‐9, *R* ^2^ = .45, Fig. S2). Aggregate abundance was 11.3 × *d* ^1.9^ (Fig. 1, converted to radius, see below for explanation), where *d* is aggregate diameter in millimeters. Pooling data reduced the *R* ^2^ but accounted for variation in the relationship across sampling dates, providing the most general estimate of this relationship given the spatial and temporal limits of the available data. The area under the continuous function was integrated for 10 equally sized bins (on the log scale) from 0.2 to 20 mm diameter (macroaggregates) and an additional bin for 0.05–0.2 mm diameter (microaggregates). Aggregate size has generally been reported by diameter in field data, but from this point forward, we will use aggregate radius, which is the principal convention in the experimental literature. Both metrics are actually effective measures as aggregates are often not spherical. Microaggregates are large enough to be captured with near 100% efficiency, but too small to be regularly counted and reported in the literature. The proportion of the total area occupied by each size class was then used to randomly draw a collection of 500,000 aggregates in the same proportions to use in the feeding simulation. The simulations examine a range of aggregate abundances from 50 to 500/L that encompasses the commonly observed range of normal variation in coastal systems (Lyons et al., 2007; Newell et al., 2005; Simon et al., 2002).

### 2.5 Pathogens on aggregates

The number of pathogen cells on an aggregate was estimated using a variation of the stochastic bacteria‐aggregate model in Kramer et al. (2013). The code is archived on Dryad (doi: 10.5061/dryad.m56c1). This model used a colonization–birth–death process to simulate bacterial colonization, attachment, detachment, and reproduction, as well as predation of bacteria by flagellates and ciliates, on aggregates of different sizes that form in different concentrations of unattached pathogenic bacteria. The model was originally parameterized for the average behavior of the marine bacterial community (Kiørboe, 2003; Kramer et al., 2013), so in the present study the pathogen detachment rate was decreased by 90% to approximate a vibrio bacterium, many of which are known to be strongly particle‐associated (e.g., Preheim, Timberlake, & Polz, 2011). These “sticky” bacteria result in higher average abundances of pathogen than reported in Kramer et al. (2013), and are a hypothesis for why *Vibrio* enrichment factors on aggregates generally are high (Froelich et al., 2013; Lyons et al., 2007, 2010; Worden et al., 2006). We are unaware of any direct measurements of colonization or vital rates on aggregates for any pathogen species that would allow empirical parameterization. This modified model was then simulated for macroaggregates with radii equal to the midpoints of the 10 bins between 0.1 and 10 mm, and unattached pathogen concentrations of 10, 100, 1000, and 10,000 cells/ml. These sizes and concentrations were chosen to match the range of the model in Kramer et al. (2013) and the range of concentrations of pathogenic bacteria in seawater (2–40,000 cells/ml, Wright et al., 1996; Lyons et al., 2007; Travers et al., 2009; Vezzulli et al., 2010; Plano et al., 2013). The upper bound for pathogenic concentrations, ca. 10^6^ cells/ml, has been reported for the sediment–water interface (Travers et al., 2009) and in a sewage spill scenario (Shchegolkova et al., 2016). The total concentration of all bacterial species, of which the focal pathogen was a subset, was assumed constant at 10^6^ cells/ml (Kiørboe et al., 2003; Kramer et al., 2013).

To obtain a distribution of bacterial abundance for each unattached pathogen concentration on aggregates of each size, the stochastic realizations of the bacteria–aggregate dynamical model were sampled by randomly selecting a time point in the 1‐week lifetime of the ~1,000 simulated aggregates. The aggregates consumed by the simulated oysters were then randomly drawn from this distribution. This procedure resulted in a combination of pathogen abundances from aggregates of different ages and histories.

The pathogen abundance on microaggregates (radius 0.025–0.1 mm) was taken to be equivalent to that of the smallest macroaggregate size class. We did not separately simulate these aggregates because the physics and biology observed for macroaggregates and implemented in the bacteria–aggregate model are unstudied at the smaller aggregate scales (Kiørboe, 2003). The lack of empirical data made this naïve extrapolation seem to be the least risky while still accounting for this important class of particles; microaggregates make up ~70% of the aggregates in the estimated size distribution (Fig. 1).

### 2.6 Simulations

The feeding model was implemented in NetLogo (Wilensky, 2012) and R (R Development Core Team 2014), and code is archived on Dryad (doi: 10.5061/dryad.m56c1). Oysters were treated as fixed grid cells in a 21 × 21 grid that initially contained no pathogen cells. The total number of aggregates consumed by all oysters in a time step was determined from the macroaggregate concentration, with microaggregates then added in the proper ratio. Microaggregates were handled in this way so as to report aggregate concentration consistently with the literature, as microaggregates are not counted or reported in most studies. Random variation in number of aggregates consumed and the number of pathogen cells on those aggregates then entered the model in two ways. First, the number of aggregates consumed was chosen from the stochastic collection of aggregates described above, with sizes distributed as estimated from Shanks (2002), and pathogen abundances distributed as estimated by the stochastic model of pathogen on aggregates (Kramer et al., 2013). Second, these aggregates were randomly distributed over the grid, then consumed by the oyster in the corresponding cell. Each oyster was also assumed to consume a fixed number of unattached cells depending on concentration of pathogen in the water. A total of 176 simulations were run for the combinations of 10 concentrations of macroaggregates (50–500 aggregates/L, increment by 50) and four unattached pathogen concentrations (Table 1) with the oysters feeding continuously for 4 days.

### 2.7 Model variations

To explore the importance of model assumptions, three alternative models were also simulated. (1) A model with “average” bacteria (Kramer et al., 2013). This model uses the empirically measured detachment rate for the bacterial community from Kiørboe et al. (2003), instead of the “sticky” vibrio‐type detachment rate, and is otherwise the same as the full model. (2) A model without microaggregates. Microaggregate concentrations are often unmeasured in the field, and very small aggregates have low pathogen abundances (Kramer et al., 2013). (3) A model with lower clearance rates. Clearance rate was set to 50% of the full model, representing a reasonable estimate for smaller oysters or larger oysters exhibiting variable feeding.

### 2.8 Analysis of simulated data

The abundance of pathogen cells in the oysters was sampled from the simulation output at 12 hr intervals. The means and the 2.5 and 97.5% quantiles of the unattached and aggregate‐associated pathogen from each set of 176 simulations were calculated at each time point. The maximum abundance of pathogen cells was taken to be the largest value of mean abundance in the time series; in most cases, this occurred at the end of the time series.

## 3 Results

**Figure 2:**
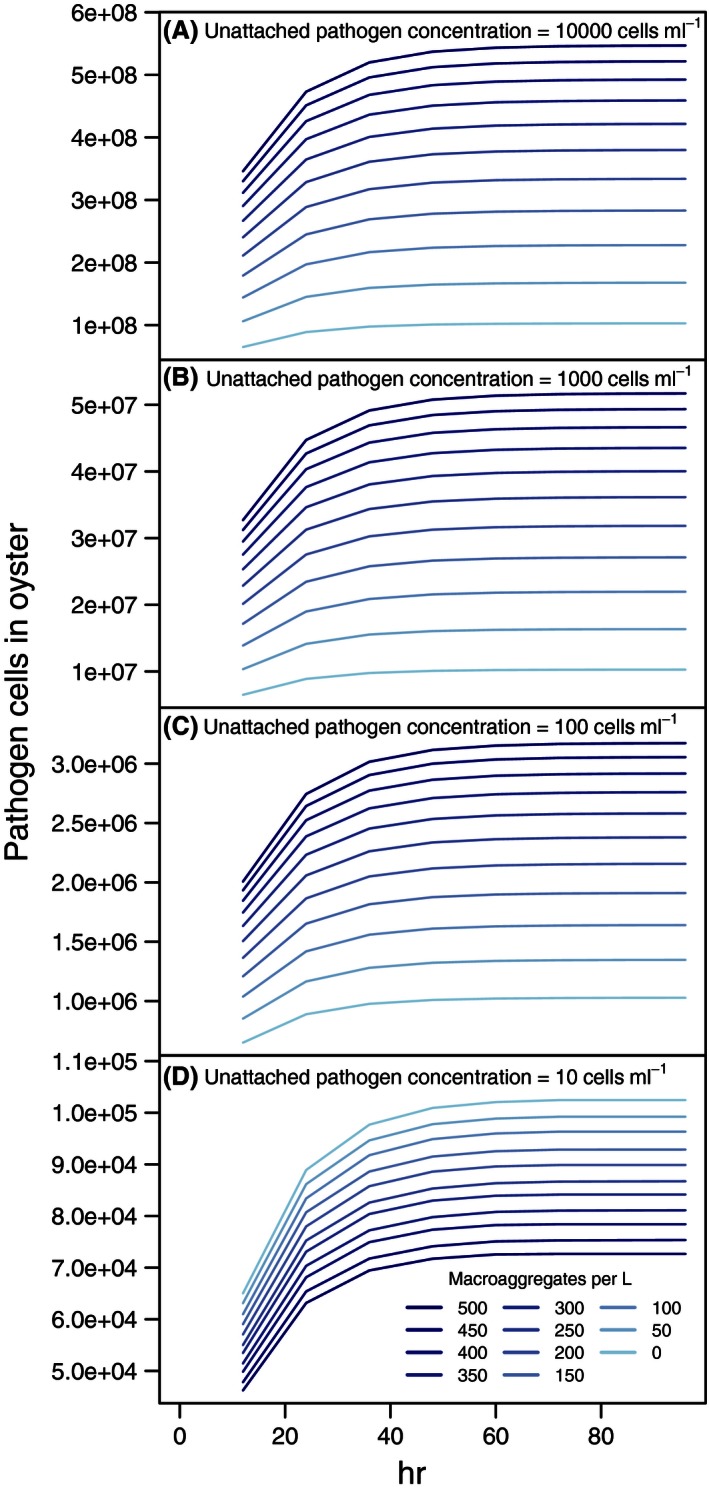
Accumulation of hypothetical “sticky” pathogen in an oyster. The abundance of cells is shown over a simulated period of 4 days. Results are shown for unattached pathogen cell concentration in water of: (A) 10,000 cells/ml (1% of total bacterial cells in water), (B) 1,000 cells/ml, (C) 100 cells/ml, and (D) 10 cells/ml. Unattached pathogen concentration and aggregate concentration were assumed constant at each level for the entire time period. The estimated number of pathogen cells in the oyster begins to plateau after 2 days. At the lowest unattached pathogen concentration, the pathogen cells in the oyster are lower at higher aggregate concentration due to the relationship between aggregates concentration and pseudofeces production. Standard error bars are sufficiently small as to not be visible

The model predicted rapid pathogen accumulation in oysters, with abundances after the first 12 hr ranging from 20% to 50% of the maximum abundances (Fig. 2). Accumulation leveled off by 72 hr, the time at which pathogen ingestion was nearly balanced by pathogen loss via digestion and pseudofeces production, and reached near maximum by 96 hr. The asymptote was approached most quickly for scenarios involving low concentrations of aggregates and high concentrations of unattached pathogens (Fig. 2A). Higher aggregate concentrations led to a higher rate of pathogen accumulation, but increased the time to approach the asymptotic maximum because the maximum number was higher (Fig. 2A–C). The notable exception to these trends was that aggregates decreased the number of pathogen cells ingested by oysters at low unattached concentrations (Fig. 2D), a result of increased pseudofeces production leading to rejection of unattached pathogen. Under conditions of low unattached pathogen concentrations, there were few pathogens on aggregates because colonization was balanced by extinction.

**Figure 3:**
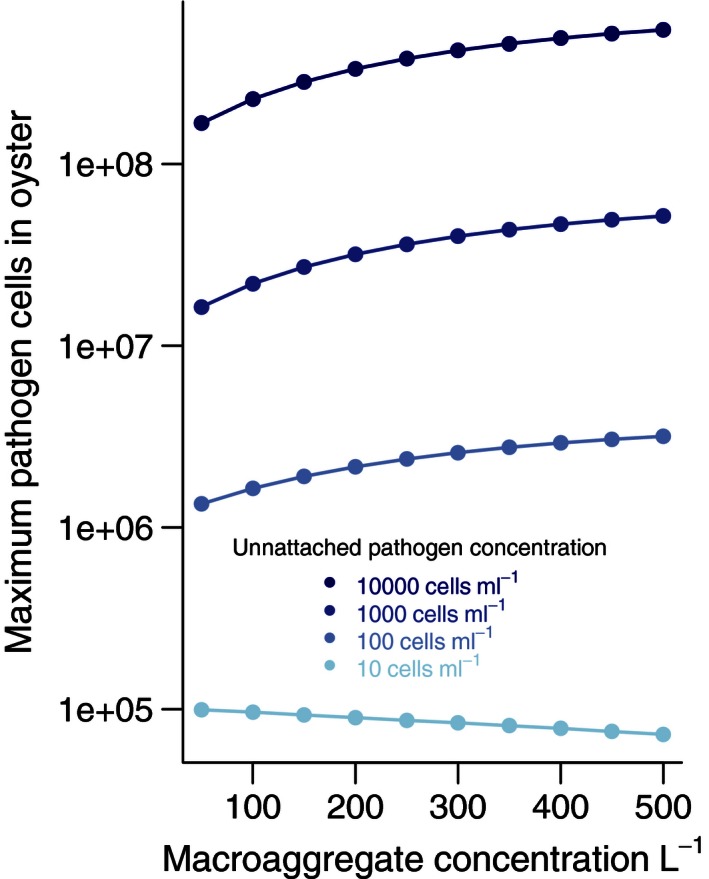
Effect of unattached, “sticky” pathogen concentration and aggregate concentration on pathogen accumulation in an oyster. Estimated pathogen load is shown after 4 days for four unattached pathogen concentrations (different colors) at 10 aggregate concentrations. Pathogen load increases noticeably with aggregate concentration at high ambient concentrations. At the lowest unattached pathogen concentration, the load decreases with aggregate concentration, due to the modeled increase in pseudofeces production. Parameters were assumed constant over the 4 days. This plot is a simplified representation of the maximum values from Figure 2, emphasizing the effects of aggregate and pathogen concentration. Standard error bars are sufficiently small as to not be visible

The average maximum pathogen concentration in the oysters was highly correlated with both the concentration of unattached pathogen and aggregates (Fig. 3). Unattached pathogen concentration was the most influential, with 10‐fold increases leading to 10‐fold increases in pathogen accumulation (Fig. 3). By comparison, 10‐fold increases in aggregate concentration led to twofold to threefold increases in the number of pathogen cells in the oysters. For the lowest unattached concentration, however, the average maximum pathogen abundance in oysters declined, due to the increase in pseudofeces production that resulted from increased concentration of aggregates in the water. This result suggests that bistability arises in the system from low colonization rates and high extinction rates when pathogens are sparse (Kramer et al., 2013). The 95% quantiles around these average maximum abundances were small (invisible on Fig. 3), as a result of the large volume of water from which oysters cleared aggregates and pathogens over the hours of simulation, averaging out random variation.

**Figure 4:**
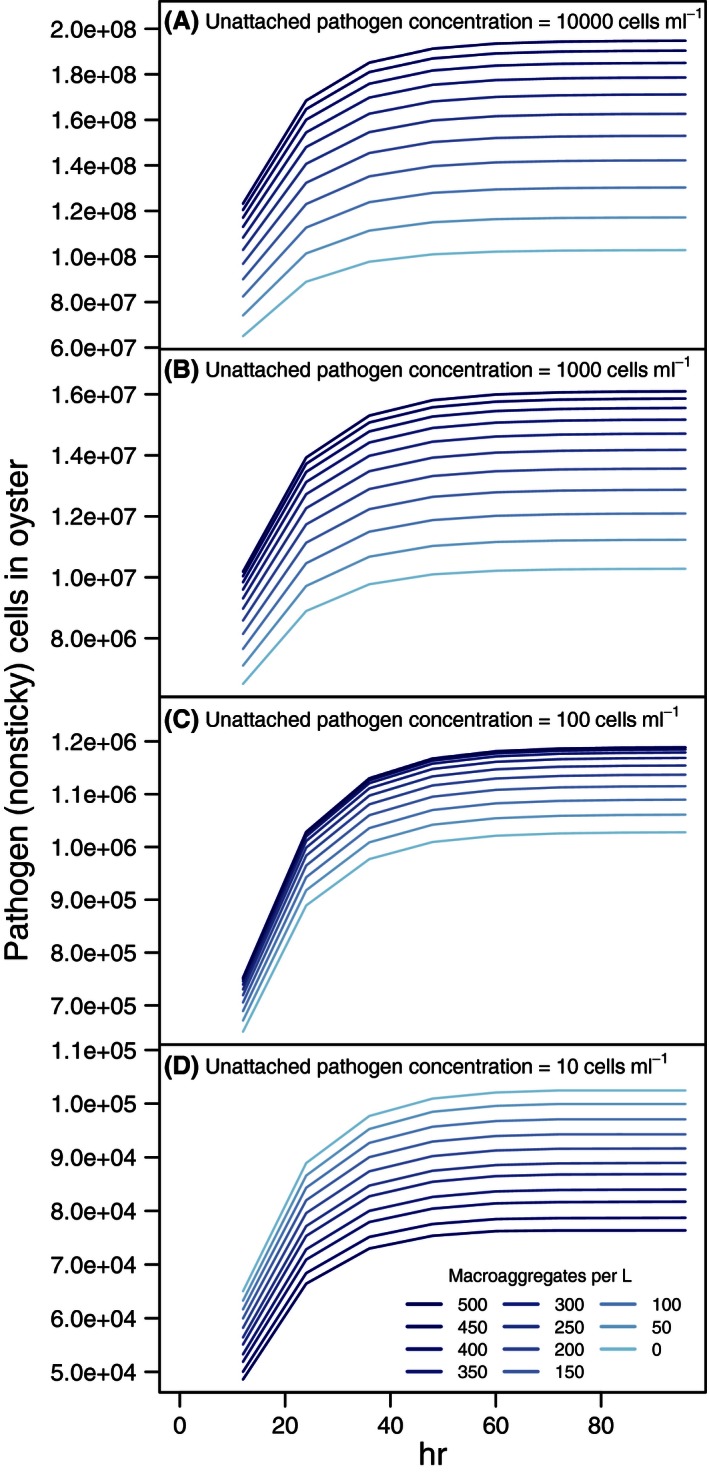
Accumulation of hypothetical pathogens in an oyster estimated from a model with average bacteria. These “nonsticky” bacterial cells have no preference for aggregates. The abundance of cells is shown over a simulated period of 4 days. Results are shown for pathogen cell concentration in water of: (A) 10,000 cells/ml (1% of total bacterial cells in water), (B) 1,000 cells/ml, (C) 100 cells/ml, and (D) 10 cells/ml. Unattached pathogen concentration and aggregate concentration were assumed constant at each level for the entire time period. The estimated number of pathogen cells in the oyster begins to level off after 2 days. At the lowest unattached pathogen concentration, the pathogen cells in the oyster are lower at higher aggregate concentrations due to the relationship between aggregate concentration and pseudofecal production. Standard error bars are sufficiently small as to not be visible

When the detachment rate of the average “nonsticky” bacterial species was substituted for the rate associated with “sticky” vibrios, accumulation in oysters at high concentrations of unattached pathogens reached a maximum of less than half that of the sticky pathogen at similar bacteria and aggregate concentrations (Fig. 4). At an unattached pathogen concentration of 10 cells/ml, the results were almost identical as the sticky pathogen because aggregates contributed very little to pathogen accumulation. At the intermediate concentration of 100 cells/ml, aggregate concentration also had very little influence on accumulation of pathogen in the oyster, because there were fewer pathogens on the aggregates, a result that contrasts with the simulation using the sticky pathogen. In all scenarios, the average pathogen load reached its asymptotic value more quickly (ca. 48 hr). The results from the nonsticky model contrast with the removal of microaggregates, which had very little effect on the average concentration of pathogen in oysters (Fig. S3). Reduction of clearance rate had the anticipated effect of halving the amount of pathogen in the oyster while having little effect on the relative rate of accumulation (Fig. S4).

**Figure 5:**
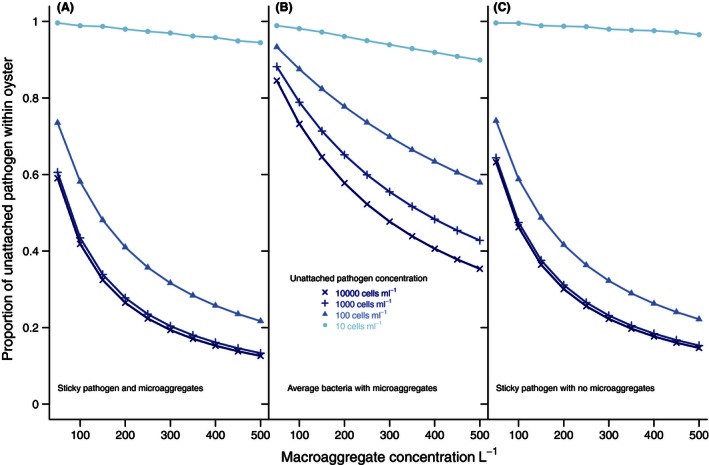
Proportion of pathogen load attributable to consumption of unattached cells vs. cells within aggregates. The pathogen load estimated to be in oysters after 4 days can be partitioned into cells cleared from suspension as unattached vs. those attached to aggregates. These are shown for (A) the full model (“sticky” pathogen) and for versions with (B) nonsticky pathogen and (C) without microaggregates. Importance of aggregates accelerates with increased unattached pathogen concentration, and asymptotes at high aggregate concentrations with the three higher pathogen concentrations. Microaggregates have little influence on the proportion of pathogen from the aggregates, while pathogen preference for aggregates (“stickiness”) has a large effect. Standard error bars are sufficiently small as to not be visible

The contribution of aggregates to the average maximum pathogen load of oysters was highly dependent on the concentration of unattached pathogens (Fig. 5). When low (10 cells/ml), essentially all of the pathogen accumulation in an oyster came from unattached cells. When unattached pathogen concentration in the water increased, aggregates contributed significantly to pathogen load in oysters. For example, at unattached concentrations of 100 cells/ml, the percentage of pathogens in oysters attributed to unattached cells decreased from 75% to 22% as the macroaggregate concentration increased from 50 to 500/L (Fig. 5A). At even higher unattached pathogen concentrations of 1,000 and 10,000 cells/ml, aggregates had a larger contribution, but the effect leveled off and became nearly identical for the two concentrations (Fig. 5A,C). The model in which pathogen cells detached at average rates (nonsticky pathogens as in (Kramer et al., 2013)) showed much less influence of aggregates (Fig. 5B). Conversely, eliminating microaggregates decreased the contribution of unattached cells only slightly (Fig. 5C). For all models, the effect of increasing the concentration of aggregates in the water was small and nearly linear at the lowest unattached pathogen concentration, and then increased nonlinearly at the higher concentrations of unattached pathogens.

## 4 Discussion

Our modeling study indicates that aggregates can play an important role as a transporter for bacterial uptake by bivalves such as oysters. This outcome matches empirical studies that have shown increased numbers of bacteria, including pathogens, in oysters when aggregates are present in the water column (Froelich et al., 2013; Kach & Ward, 2008). At the same time, the results emphasize that unattached bacteria contribute significantly to bacterial uptake even in the presence of aggregates. The model makes important additional contributions; first, by characterizing how the relative contributions of unattached and aggregate‐associated pathogens affect concentrations of pathogens in oysters, and second, by characterizing how results depend on the bacterial concentration in the water and the abundance of aggregates. When pathogenic bacteria were at a low concentration in the water, aggregates did not increase bacterial uptake because aggregates had few bacteria associated with them. At the same time, unattached cells contributed a sizable proportion of bacterial uptake even at high aggregate concentrations (e.g., 300 aggregates/L). In the context of human and wildlife health, those conditions under which aggregates were most contributory (greater concentrations of unattached pathogen) were also the conditions under which pathogen load in shellfish quickly reached large absolute magnitudes. Therefore, when abundance of pathogens in bivalves already was substantial in the absence of aggregates, aggregates’ presence accelerated the uptake of pathogens. The result was large increases in pathogen abundance in bivalves. The relationship between unattached pathogen concentration and the contribution of aggregates to pathogen load in oysters was highly nonlinear, caused primarily by the nonlinear relationship between aggregate size and bacterial abundance on aggregates at low pathogen concentration (Kramer et al., 2013). The concentrations of macroaggregates used in these simulations are in line with those reported in nature (Simon et al., 2002), suggesting that these scenarios can be tested empirically, and if borne out, possibly be incorporated into oyster harvesting operations.

Under constant conditions, simulated pathogen concentrations in oysters increased rapidly and reached a near steady‐state abundance after 2–3 days. While pathogen and aggregate concentrations can be variable, the effect of short‐term variability is reduced by the high potential feeding rates of bivalves. At the lowest pathogen and aggregate concentrations considered, the simulated oysters would have ingested tens of thousands of pathogen cells following 12 hr of constant feeding. Because this is the same timescale as gut clearance, significant periods without feeding will greatly reduce this abundance, as will lower rates of particle clearance. The approach to steady state is delayed for higher concentrations of unattached pathogens and aggregates. One unexpected model prediction is that under high levels of aggregates and low concentrations of pathogenic bacteria, oysters may accumulate fewer pathogens than in the absence of aggregates. This outcome is a result of increasing pseudofeces production leading to rejection of unattached bacteria and aggregates which contain few bacteria and themselves contribute little to pathogen uptake. If shown to hold under natural conditions, this result has interesting implications for risk of vibrio infection following consumption of shellfish. When vibrio concentrations in the water column are low, as is generally the case, the model suggests aggregates somewhat reduce uptake of vibrio by shellfish. When vibrio concentrations increase, for example, during warm‐water conditions optimal for their growth, aggregates may then play an important role in their uptake by shellfish.

As the first attempt to understand the contributions of aggregate‐associated and unattached bacteria to concentrations of pathogenic bacteria in oysters, the model proposed here was necessarily greatly simplified. Important environmental variation such as variable feeding rates, spatial gradients of aggregate availability as a result of water currents or depth, and tidal cycles were not explicitly simulated, but the simulations of different aggregate abundances and of reduced feeding rate give an initial indication of how such variation will influence results. The tight quantile bounds on the results indicate that stochasticity in the sizes of aggregates consumed and their individual bacterial load evened out over several hours of constant filtering. Whereas consumption of unattached bacteria was not modeled stochastically, we believe that this unaccounted source of intrinsic noise would not constitute a significant source of additional variation, given the large clearance volumes involved. Introducing additional sources of variation or focusing on bivalves with lower clearance rates, as a result of smaller body size or species‐specific behavior, however, would increase the importance of stochasticity. Nevertheless, the model suggests that such variation will be unimportant for the potential effects on animals or humans consuming contaminated shellfish, unless minimum infectious dose and bacterial concentration in the water are both very low. Rather, variation in the load of pathogens accumulated by oysters in nature more likely comes from differences in the feeding rates of individuals or microhabitat variation (e.g., position on a reef), rather than chance consumption of individual aggregates.

The relationships reported here depend on four assumptions made to increase the realism of the model, but for which direct empirical support is limited. First, the rate of loss of the pathogen from the aggregates was assumed to be much lower than the empirically estimated parameter for the bacterial community as a whole (Grossart, Hietanen, & Ploug, 2003; Kiørboe et al., 2003; Kramer et al., 2013). The rate of loss, which integrates detachment and nonpredation mortality, was lowered to approximate pathogenic bacteria known to be attracted to and to thrive on marine aggregates, namely many species of vibrios (i.e., sticky pathogens). The rate of loss has not been measured for any individual bacterial species, but indirect empirical studies suggest that aggregates are more enriched in some classes of heterotrophic bacteria than predicted for the average bacteria by Kramer et al. (2013). Importantly, the mechanism of this enrichment has not been directly measured and the design of the empirical studies precludes direct comparison of enrichment factors and simulated abundances (Froelich et al., 2013; Lyons et al., 2007, 2010). Therefore, the model reflects species with a propensity to associate with aggregates to a greater or lesser extent. Scenarios with a nonsticky pathogen illustrated that not only is accumulation reduced, but the importance of aggregates can also be reduced at lower unattached pathogen concentrations. Predictive modeling would greatly benefit from more measurements on individual bacterial species and their association with aggregates.

A second assumption concerns the model's inclusion of microaggregates. It is well understood that aggregates with radii 0.025–0.1 mm are more abundant than macroaggregates, but we found no studies measuring size distributions across the entire size spectrum. Aggregates in this size range are expected to be captured at high efficiencies (Ward & Shumway, 2004), so treating their retention similarly to macroaggregates is well supported. However, the population dynamics of bacteria and flagellates on aggregates of this size are unstudied, forcing us to treat microaggregates as similar to their nearest neighbors, aggregates of 0.1 mm radius.

The third assumption was that of reasonable extrapolation from the datasets used to connect components of the model. Because of the limited published data on (1) the size distribution of aggregates, (2) the relationship between aggregate concentration and SPM density, and (3) the effect of aggregate concentration on pseudofeces production, it was necessary to extrapolate at the boundaries of the best available published data. We extrapolated at the upper and lower ends of the aggregate size distribution, and note that this relationship was based on a specific location and year (Shanks, 2002). Similarly, the connection between aggregate concentration and total SPM also was based on one location (Shanks, 2002). This relationship is likely dependent on the types of suspended particles available in the water column and therefore changes with environmental conditions (e.g., Newell et al., 2005). We also extended the upper end of the pseudofeces production function in order to match the sampled data on aggregate densities and SPM. These extrapolations clearly limit the direct transferability of the quantitative results, but given the focus on broader patterns and constructing a usable model, are unlikely to alter the conclusions. These results would not hold, however, were the extrapolated relationships to become highly nonlinear beyond the limits of the published data. Indeed, the necessity of making such extrapolations and assumptions emphasizes the need for additional field measurements of aggregate and SPM concentrations, and their influence on feeding processes of oysters. Such data would allow tests of the extrapolations’ validity and if applicable, enable application of the model to different locations and environmental conditions.

Finally, we assumed that all pathogens were treated in a similar manner by the feeding organs of the oyster (e.g., gills, labial palps), and had the same likelihood of rejection regardless of whether they were captured unattached or associated with an aggregate. This assumption was based on studies which demonstrate several key points regarding how bivalves feed on aggregated material. First, the ciliary activity on the gills and palps of bivalves can disrupt and separate particle aggregations into smaller particle masses and, perhaps, individual cells (Ward, 1996). Second, oysters and other bivalves ingest significantly more bacteria and bacteria‐size microspheres when the particles are incorporated into aggregates compared to when they are delivered freely suspended (Froelich et al., 2013; Kach & Ward, 2008). Therefore, our assumption that pathogens within an aggregate and unattached pathogen cells have the same chance of being ingested or rejected following capture is reasonable. If unattached pathogens are less likely to be rejected in pseudofeces, it would result in an undetectable effect of aggregates when bacteria are at their lowest concentration in the water, rather than the slightly negative relationship predicted by the model. This scenario would also slightly increase the proportion of pathogens accumulated by the oyster that originated as unattached cells at higher concentrations of unattached pathogen.

The model developed here provides a mechanistic framework for understanding bacteria–aggregate–bivalve interactions. Accordingly, it identifies multiple directions for improving our appreciation of these relationships. Here, we have explored the importance of bacterial associations with aggregates and microaggregates, but other aspects likely to have similarly large influences on pathogen accumulation could be included in future models. Such factors include how different groups of bacteria are digested, interactions with the resident bacterial community, species‐specific bacterial taxis toward aggregates, and the relationship between aggregate concentration and pseudofeces production. This model provides a platform for studying the effects of these processes and, given additional empirically measured parameters, could provide quantitative predictions of pathogen accumulation in shellfish. Because the basic mechanisms of particle capture are similar among suspension‐feeding bivalves, the qualitative effects of variation in unattached pathogen and aggregate concentrations are likely to hold across most species (Kach & Ward, 2008). To put the simulated pathogen accumulation in context, measured levels of *Vibrio vulnificus* are often >1 × 10^4^ cells/g oyster tissue (Morris & Acheson, 2003), whereas low risk status is set at <30 cells/g (World Health Organization 2005). It is important to note that loads needed to induce sickness differ between pathogens and often depend on the presence of virulence genes and human host immune status (Froelich & Noble, 2016).

These results constitute an advance in our comprehension of the conditions under which aggregates can be transporters of pathogens into bivalves. Notably, a “worst‐case” example, at least for human consumers of shellfish, would be a scenario in which high concentrations of pathogens were introduced to the water column during a short period of time (i.e., storm events, septic overflow, farm runoff). It is clear that the role of aggregates depends substantially on particular characteristics of the pathogen such as affinity for aggregates, high growth rate on aggregates, or species‐specific dynamics in the gut and tissues of the shellfish. In fact, even strain‐level differences can be important (Froelich, Ringwood, Sokolova, & Oliver, 2010). In general, unattached cells are likely an important source of pathogens under most environmental conditions, but aggregates also contribute to pathogen accumulation to a greater or lesser extent (ca. 80%–10%). One possible counterexample would be if aggregates act as reservoirs after unattached cells have been removed from the water column (Worden et al., 2006). Aggregates, however, often settle out of the water column faster than individual cells because of their larger size, so the rate of removal of the free cells would need to be very high to shift the predominant source of accumulation to aggregates. These results indicate a need for species‐specific investigation to determine whether aggregates can initiate the risk of contaminated shellfish or will only act as an amplifier of pathogen accumulation when the risk is already high.

## Data Availability

Code and data available at Dryad repository: http://dx.doi.org/10.5061/dryad.m56c1.

## Funding Information

Directorate for Biological Sciences, (Grant/Award Number: 0914347, 0914429, 0914459).

## Conflict of Interest

None declared.
